# The effect of therapeutic artistic activities on anxiety and psychological well-being in adolescents with type 1 diabetes: a randomized controlled study

**DOI:** 10.1007/s00431-025-06237-y

**Published:** 2025-06-03

**Authors:** Ozden Gocen, Cigdem Sari Ozturk

**Affiliations:** 1 Goztepe Prof. Dr. Suleyman Yalcin City Hospital, Pediatric Intensive Care Unit, Istanbul, Turkey; 2 Pediatric Nursing Department, Gazi University Nursing Faculty, Ankara, Turkey

**Keywords:** Adolescent, Anxiety, Art, Diabetes, Therapeutic, Well-being

## Abstract

A parallel-group, randomized controlled design was implemented in this study to examine the effectiveness of therapeutic art activities for adolescents with type 1 diabetes. The study was conducted at Ankara Bilkent City Hospital between November 2023 and August 2024 with 72 adolescents aged 9–18 (36 intervention, 36 control). The intervention group participated in 6 weeks of therapeutic art activities, while the control group received standard care. Therapeutic artistic activities included Zentangle, mandala painting, third-person drawing techniques, and the awareness of four-leaf clover. Sessions were held one-on-one via WhatsApp. The State Anxiety Inventory and Psychological Well-Being Scale were administered at the beginning and end of the study. After the therapeutic program, the mean state anxiety score of adolescents in the intervention group (32.19 ± 2.75) was significantly lower than in the control group (45.13 ± 4.84). Additionally, at the end of the program, the mean psychological well-being score of adolescents in the intervention group (48.52 ± 5.30) was significantly higher than that of the control group (33.41 ± 9.83) (*p* < 0.05).

*Conclusion*: It was concluded that therapeutic art activities were effective in reducing anxiety and improving psychological well-being in adolescents with diabetes. It is recommended that therapeutic art activities be integrated into the care programs for children with diabetes.

*Trial registration*: ClinicalTrials.gov Identifier: NCT06595173. Registered on September 11, 2024.

**Table:** 

**What is Known:** • *Diabetes diagnosis affects well-being and anxiety levels of adolescents.* • *Therapeutic programs are needed for adolescents with diabetes.*
**What is New:** • *Therapeutic artistic activities should be planned by considering adolescents’ developmental characteristics.* • *The combination of therapeutic cognitive and artistic activities is a program with a high impact size that holistically affects the concept of well-being of adolescents with diabetes.*

**What is Known:**

• *Diabetes diagnosis affects well-being and anxiety levels of adolescents.*

• *Therapeutic programs are needed for adolescents with diabetes.*

**What is New:**

• *Therapeutic artistic activities should be planned by considering adolescents’ developmental characteristics.*

• *The combination of therapeutic cognitive and artistic activities is a program with a high impact size that holistically affects the concept of well-being of adolescents with diabetes.*

## Introduction

Type 1 diabetes mellitus (T1DM) is a chronic condition frequently diagnosed during childhood. According to the International Diabetes Federations (IDF), an estimated 8.75 million individuals worldwide lived with T1DM in 2022, of whom 1.52 million were under 20 [1]. The same report indicates that in Türkiye, 143,396 individuals across all age groups have been diagnosed with type 1 diabetes, with 29,000 of them being younger than 20 years old [1]. These statistics underscore the prevalence of T1DM in childhood, with adolescence being recognized as a particularly challenging phase for diabetes management [2].

The presence of diabetes can elevate anxiety levels and adversely impact psychological well-being, particularly in adolescents [3]. Research has demonstrated that type 1 diabetes affects physical and psychological well-being [4, 5]. The chronic nature of the disease, the complexities of disease management, the necessity of daily insulin administration, and the perceived sense of difference from peers may contribute to psychological distress among adolescents with diabetes [4]. Given the importance of peer relationships during this stage, some adolescents may conceal their condition or delay treatment adherence [6], potentially leading to increased anxiety and social withdrawal [7].

Comprehensive interventions are essential to support the mental health of adolescents with diabetes. Therapeutic artistic activities have been identified as a potentially practical approach for mitigating the psychological impact of diabetes on adolescents [8]. Creative activities such as drawing, painting, and mask-making significantly alleviate the stress associated with treatment processes [9, 10]. As a result, artistic activities can be integrated into anxiety management strategies and overall well-being enhancement [11].

Artistic activities reduce trauma-related symptoms in children [12] and improve mood and blood glucose levels in individuals with diabetes [7]. Art therapy also enhances psychological well-being and a sense of meaning in young adults with diabetes [13]. Supporting mindfulness practices such as breathing exercises and muscle relaxation [14] with cognitive techniques such as the mindfulness clover approach [15] supports resilience and emotional healing.

The existing literature highlights limited studies that integrate artistic activities with cognitive techniques in therapeutic programs [16, 17]. Such interventions have primarily been utilized to enhance resilience among young individuals [18] and to promote mental well-being within school-based programs [19]. However, no studies have been identified that specifically apply therapeutic artistic activities to address anxiety and psychological well-being in adolescents with diabetes. While some interventions have individually utilized artistic or mindfulness-based techniques in adolescents with chronic illnesses [8–11], no studies have integrated these approaches into a comprehensive therapeutic program tailored for adolescents with type 1 diabetes mellitus. This study is novel in its design, combining Zentangle and mandala drawing, third-person communication, and the four-leafed awareness clover method, and it delivers these sessions individually through a mHealth platform (WhatsApp). This integrated, developmentally appropriate approach addresses the emotional and contextual needs of adolescents with diabetes. Given this gap, this study aims to contribute significantly to the literature by examining the effects of therapeutic artistic activities on adolescents with type 1 diabetes. The increasing recognition of the need for holistic approaches that address not only the physical but also the psychological well-being of individuals with chronic illnesses, such as diabetes, underscores the relevance of this research.

## Hypotheses of the study

H_1_._1_: The anxiety levels of the intervention group participating in therapeutic artistic activities will be significantly lower than those of the control group.

H_1.2_: The psychological well-being levels of the intervention group participating in therapeutic artistic activities will be significantly higher than those of the control group.

## Methods

### Study design and population

**Fig. 1:**
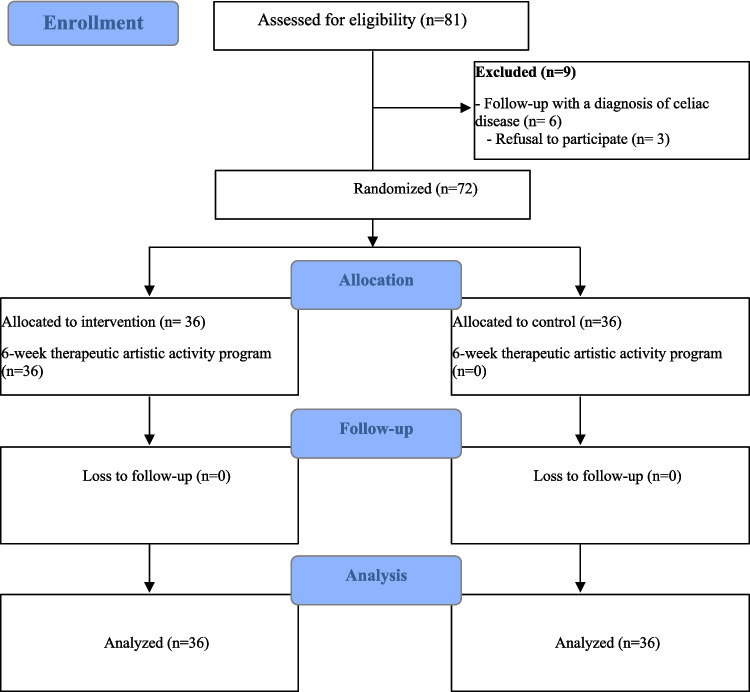
Consort flow diagram of the study

This single-blinded, parallel-group randomized controlled trial was conducted at the Pediatric Endocrinology Outpatient Clinic of a major hospital in Türkiye’s capital between November 20, 2023, and August 15, 2024. Findings followed CONSORT-Outcomes 2022 guidelines [20] (Fig. 1), and the protocol was registered on ClinicalTrials (NCT06595173).

The study included adolescents with type 1 diabetes followed at the Pediatric Endocrinology Outpatient Clinic of Ankara Bilkent City Hospital between November 20, 2023, and August 15, 2024. The required sample size was calculated using G*Power 3.1.9.7 software based on an effect size of *d* = 0.95, a significance level (*α*) of 0.05, and a statistical power (1 − *β*) of 0.95 for two independent groups. This analysis indicated that a minimum of 60 participants (30 per group) was necessary to detect a statistically significant difference. To account for potential dropout, 72 adolescents were enrolled and equally randomized into the intervention (*n* = 36) and control (*n* = 36) groups using stratified block randomization. A post hoc power analysis confirmed the study’s sufficient statistical power (100%) based on the observed effect sizes (*d* = 3.29 for anxiety; *d* = 1.91 for psychological well-being).

### Inclusion criteria

Participants were included if they:

- Were adolescents aged 9–18 year with type 1 diabetes diagnosis for at least 6 months
- Had internet access
- Had no additional medical or psychological conditions
- Did not use psychoactive medications or receive ongoing psychiatric treatment
- Provided written informed consent from a parent or guardian

### Exclusion criteria

Participants were excluded if they:

- Used an insulin pump
- Experienced a major life event in the past 6 months (e.g., migration, bereavement, divorce)
- Engaged in other mind–body practices (e.g., yoga, guided imagery)
- Withdrew voluntarily from the study
- Had a break of more than 2 weeks in the intervention program

### Randomization and prevention of bias

To ensure methodological rigor and minimize bias, the randomization process was carefully structured. After receiving general information about the study from the outpatient clinic nurse, adolescents who agreed to participate, along with their families, completed the Descriptive Characteristics Form and the pretest assessments. Participants were not informed of their group allocation to maintain blinding.

#### Randomization process

Following the completion of pretests, participants were stratified based on age (9–13 and 14–18 years) and gender (male and female) before being assigned to either the intervention or control group using a stratified block randomization method.

To further mitigate bias, a statistical expert independent of the research team generated randomized block allocations for the intervention and control groups using the www.randomizer.org tool. Twelve block combinations were created, with 18 adolescents assigned to each stratum. Since an insufficient number of participants were available in the 14–18 age group, an additional participant from the 9–13 age group was included to maintain balance. This adjustment ensured the integrity of the randomization process. A total of 72 adolescents (36 in the intervention group and 36 in the control group) were ultimately assigned. The homogeneity of the groups was verified using the chi-square test, which confirmed that there was no statistically significant difference between the groups (*p* > 0.05).

#### Bias prevention and blinding

To minimize bias and enhance the study’s methodological rigor, several blinding techniques were implemented. Random assignment was employed to prevent selection bias, with group allocation conducted by a biostatistician independent of the study. In addition to participant blinding, statistical and reporting blinding were also applied. Research data were anonymized and coded as “A” and “B” before being entered into the SPSS program by an individual uninvolved in the study. Statistical analyses were then performed based on these coded data by an independent statistician, ensuring objectivity in data interpretation. The final research report was also written in a coded format to prevent bias during reporting.

However, as the interventions were directly administered by the researcher, researcher blinding was not feasible. Despite this limitation, the implemented blinding strategies ensured a robust and unbiased research process.

### Data collection

To collect study data, three measurement tools were utilized: the Descriptive Characteristics Form, the State Anxiety Inventory for Children, and the Psychological Well-Being Scale.

#### Descriptive characteristics form

The Descriptive Characteristics Form was developed by the researchers based on existing literature [13, 16, 21]. The form consists of 25 questions, including 12 items related to sociodemographic characteristics and 13 items regarding diabetes diagnosis and treatment. The form was completed on the first day of the study after obtaining the necessary permissions from both the child and their parents. On average, participants took 5–6 min to complete the form.

#### State anxiety inventory for children

The State Anxiety Inventory for Children was originally developed by Spielberger (1973) to assess anxiety levels in children. The Turkish adaptation, including its validity and reliability testing, was conducted by Özusta (1995). The inventory consists of 20 items designed to measure the child’s current emotional state, with total scores ranging from 20 to 60, where higher scores indicate greater levels of anxiety. The Cronbach’s alpha reliability coefficient for the original scale was reported as 0.78 [22] while the Turkish adaptation demonstrated an internal consistency of 0.82 [23]. In this study, the Cronbach’s alpha coefficient was 0.81, indicating high internal consistency. The inventory was administered at the first and sixth weeks of the study, with an average completion time of 3–5 min.

#### Psychological well-being scale

The Psychological Well-Being Scale was developed by Diener et al. [24] and translated into Turkish by Telef [25]. The scale consists of eight items, rated on a 7-point Likert scale, ranging from 1 (strongly disagree) to 7 (strongly agree). Total scores range from 8 to 56, with higher scores reflecting greater psychological well-being. The Cronbach’s alpha coefficient for the original scale was 0.80, while this study found an internal consistency of 0.95, demonstrating excellent reliability. The Turkish version has also been frequently used to assess psychological well-being among adolescents in Türkiye [26, 27], demonstrating strong psychometric reliability. The scale was administered at the first and sixth weeks of the study, with an average completion time of 2–3 min.

### Intervention

#### Content of the therapeutic communicative, artistic, and cognitive activities program

The intervention program incorporated Zentangle and mandala activities as artistic activities, while the third-person technique was employed as a therapeutic communication approach during discussions related to artistic activities. Additionally, the four-leafed awareness clover technique was implemented as a cognitive activity to enhance mindfulness and self-awareness. The intervention included four structured activities, each selected for its therapeutic contribution. Zentangle and mandala coloring were used to promote relaxation, reduce anxiety, and enhance focus through repetitive, mindful drawing. The third-person technique was employed to facilitate emotional expression safely and indirectly, encouraging adolescents to reflect on their feelings without personal exposure. The four-leafed awareness clover activity was included to foster cognitive self-awareness by helping participants explore their values and inner strengths. Integrating these components allowed the program to address multiple aspects of psychological well-being: emotional, cognitive, and expressive, offering a more comprehensive intervention experience. The activities used in the study and their characteristics are shown in Table 1.

**Table 1:** Practices included in the program of therapeutic communicative, artistic, and cognitive activities and their characteristics

Elements of the program	Activity	Implementation time	Duration	Platform used	Objective
Communicative	3rd-person technique	Week 3 Week 5	10–15 min	WhatsApp	Expressing the adolescent’s feelings and thoughts without directly associating them with him/herself, thus enabling the adolescent to express himself/herself comfortably, develop empathy, and gain insight
Artistic	Zentangle	Week 2	30–40 min	WhatsApp	Reducing the adolescent’s anxiety through drawing repetitions, focusing on the moment, developing imagination, increasing well-being
	Mandala coloring	Week 4	30–40 min	WhatsApp	Creating a relaxation effect in adolescents, resting the mind and increasing self-awareness about their emotions
Cognitive	Four-leafed awareness clover	Week 2–5	15–20 min	WhatsApp	Seeing the values in the adolescent’s life, developing awareness

To facilitate adolescents’ readiness for the therapeutic artistic activity program, a Zentangle drawing video was developed. This 2-min and 37-s video was created by the researchers specifically for the study. To ensure its validity and appropriateness, the video was reviewed by three faculty members specializing in pediatric health and diseases nursing, and revisions were made based on their expert feedback before finalization. Prior to implementation, the video was shown to two healthy adolescents to assess its comprehensibility and ensure that the instructions were clear and accessible. Other therapeutic activities included in the study were conducted through individual sessions with adolescents via the WhatsApp platform, ensuring flexibility and accessibility in program delivery.

#### Implementation process of the intervention program

**Fig. 2:**
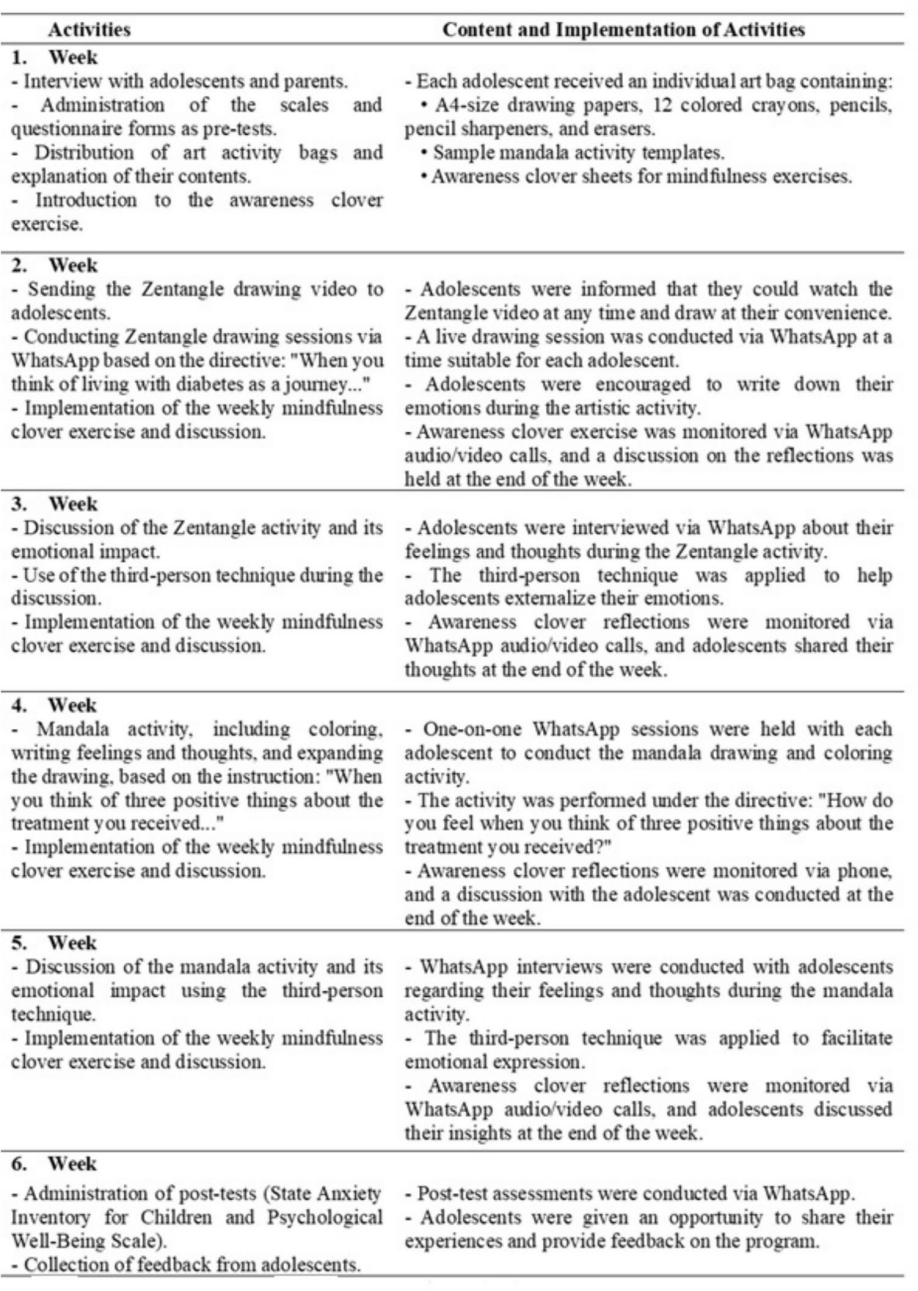
Therapeutic artistic activities and their content

Adolescents in the intervention group participated in a 6-week therapeutic artistic activities program that incorporated Zentangle, mandala drawing and painting, the third-person technique, and the awareness clover method. The program duration was determined based on adolescent developmental characteristics and prior studies, which have implemented similar interventions over 6 to 12 weeks [16, 21]. The program was structured as one individual weekly session over six consecutive weeks. Each session lasted approximately 30 to 45 min, depending on the activity. The sessions included one or more of the following: Zentangle drawing, mandala coloring, the third-person technique, and the four-leafed awareness clover activity. Details regarding the content and implementation time of each activity are presented in Table 1 and Fig. 2.

During the first week, adolescents and their families were informed about the study by a nurse at the Pediatric Endocrinology Outpatient Clinic. Those who agreed to participate completed the Descriptive Characteristics Form, the State Anxiety Inventory for Children, and the Psychological Well-Being Scale. Each adolescent received an individualized art supply bag, which included a drawing notebook, 12 colored pencils, a graphite pencil, a pencil sharpener, an eraser, mandala templates, and four leaf-clovers.

Participants were informed that the researcher would communicate via WhatsApp to schedule and conduct individual sessions at times convenient for them. The details of the therapeutic artistic activity program, including specific interventions and their implementation characteristics, are outlined in Fig. 2. To ensure intervention fidelity, all sessions were conducted by the same researcher following a standardized protocol. The sequence and content of the activities were pre-determined and followed consistently across participants, as outlined in Fig. 2. The researcher used a session fidelity checklist to ensure each intervention component was delivered as planned. Due to ethical considerations, sessions were not recorded.

Adolescents in the control group received standard hospital care without any exposure to the therapeutic artistic activities program. No additional interventions were applied to this group. To ensure consistency in data collection, the adolescents in the control group completed the pretest and posttest assessments of the State Anxiety Inventory for Children and the Psychological Well-Being Scale in parallel with the intervention group.

The intervention was delivered by the first author, a pediatric nurse with a master’s degree and clinical experience working with children with diabetes. She also holds an international certification in art therapy practice. The second author, an associate professor in pediatric nursing, holds international certifications in art therapy and mindfulness and has conducted multiple evidence-based studies on the effectiveness of art-based psychosocial interventions in pediatric care. These qualifications ensured that the intervention was delivered with professional competence and methodological rigor.

### Data analysis

Statistical analyses were conducted using IBM SPSS 25. Descriptive statistics were performed, and the Kolmogorov–Smirnov test assessed normality. Independent sample *t*-tests (between groups) and paired sample *t*-tests (within groups) were used for normally distributed data. For non-normal data, the Mann–Whitney *U* and Wilcoxon tests were applied. Effect sizes were calculated using Cohen’s *d* and classified as very small (*d* < 0.2), small (0.2 ≤ *d* < 0.5), moderate (0.5 ≤ *d* < 0.8), and large (*d* ≥ 0.8) (Cohen, 1988). The significance level was set at *p* < 0.05.

### Ethical considerations

The study received institutional (2023–20) and Gazi University ethics committee approval (03.10.2023–17/2023–1197). Written informed consent was obtained from adolescents and their families. This study was performed in line with the principles of the Declaration of Helsinki. After the study, the control group was given access to intervention materials, including art bags at the clinic and a Zentangle video via WhatsApp. No participants opted to join the program later. The study was entirely self-funded, with no external financial support. To ensure participant confidentiality and data privacy, each adolescent was assigned a unique code, and no personal identifiers were linked to the research data. WhatsApp communication was conducted using a dedicated mobile device solely for study purposes. All session records were anonymized and permanently deleted after data collection. The informed consent process explicitly explained the use of WhatsApp for scheduling and intervention delivery. These procedures were reviewed and approved by the institutional ethics committee.

## Results

### Findings related to descriptive characteristics of adolescents and their families in the intervention and control groups

Table 2 presents the descriptive characteristics of adolescents and their families. No significant differences were found between groups in adolescent age, parental age, gender distribution, or parental education levels.

**Table 2:** Descriptive characteristics of adolescents and their families in the intervention and control groups

	Intervention group (*n* = 36)			Control group (*n* = 36)		Test	
**Features**	**(*M* ± SD)**	**Min–Max**		**(*M* ± SD)**	**Min–Max**	***t***	***p***
**Adolescent age**	13.19 ± 2.56	9–17		13.22 ± 2.68	9–17	–0.044	0.96
**Mother’s age**	40.33 ± 5.46	30–51		38.41 ± 4.58	31–47	1.612	0.11
**Father’s age**	44.19 ± 6.67	31–56		41.50 ± 5.26	33–51	1.900	0.06
	***N***	***%***		***n***	**%**	***χ***^**2**^	***p***
**Gender**							
Female	17		47.22	18	50	0.814	0.500
Male	19		52.77	18	50		
**Education level of mother**							
High school and below	20		55.6	19	52.8	0.056	0.813
Undergraduate and above	16		44.4	17	47.2		
**Education level of mother**							
High school and below	17		47.2	13	36.1	0.914	0.339
Undergraduate and above	19		52.8	23	63.9		

### Findings related to diabetes treatment and the effects of the disease on adolescents in the intervention and control groups

No significant differences were found between the intervention and control groups in terms of HbA1c levels, daily calorie intake, exercise habits, hypoglycemia/hyperglycemia frequency, or the impact of diabetes on daily and school life. Although not presented in the table, no significant difference was found between the intervention and control groups regarding family support (*p* > 0.05) (Table 3).

**Table 3:** Comparison of the findings related to diabetes treatment and the effects of the disease on adolescents in the intervention and control groups

	Intervention group (*n* = 36)		Control group (*n* = 36)		Test	
	(*M* ± SD)	Min–Max	(*M* ± SD)	Min–Max	*t*	*p*
**HbA1c (last value)**	8.89 ± 2.46	4.90–16.50	8.38 ± 1.70	6.10–12.20	1.023	0.310
	***n***	**%**	***n***	**%**	***X***^**2**^	***p***
**Number of meals per day**						
> 3	26	72.2	12	33.3	1.0953	0.001
3	10	27.8	24	66.7		
**Calorie measurement**						
No	32	88.9	31	86.1	0.127	0.722
Yes	4	11.1	5	13.9		
**Regular exercise**						
Yes	21	58.3	16	44.4	1.39	0.238
No	15	41.7	20	55.6		
**Regular health check-ups**						
Yes	26	72.2	31	86.1	2.105	0.147
No	10	27.8	5	13.9		
**Development of hypoglycemia (past month)**						
Yes	29	80.6	28	77.8	0.084	0.772
No	7	19.4	8	22.2		
**Development of hyperglycemia (past month)**						
Yes	34	94.4	31	86.1	1.424	0.429
No	2	5.6	5	13.9		
**Impact on daily life**						
No	24	66.7	19	52.8	2.776	0.096
Yes	12	33.3	17	47.2		
**Impact on school life**						
No	26	72.2	19	52.8	2.904	0.088
Yes	10	27.8	17	47.2		

### State anxiety inventory and psychological well-being scale scores

Before the program, it was determined that the State Anxiety Inventory and Psychological Well-Being Scale scores of adolescents were similar in both the intervention and control groups, with no statistically significant difference between them (*p* > 0.05) (Table 4).

**Table 4:** Comparison of State Anxiety and Psychological Well-Being Scale scores of adolescents in the intervention and control groups

Scale	Intervention group (*n* = 36)		Control group (*n* = 36)			
	*X̅* ± S. S	Median [IQR]	*X̅* ± S. S	Median [IQR]	Test	Impact** size
State Anxiety Inventory						
Before the program	36.50 ± 6.89	32 [4]	37.88 ± 5.23	36 [8]	*Z* = − 1.844 *p* = 0.060	*d* = 0.23
After the program	32.19 ± 2.75	32 [4]	45.13 ± 4.84	47 [4]	*Z* = − 6.669 *p* < 0.001	*d* = 3.29
Test	*Z* = − 3.439; *p* = 0.001		*Z* = − 4.906; *p*< 0.001			
Effect size	*d* = 0.63		*d* = 1.39			
Psychological Well-Being Scale						
Before the program	39.86 ± 8.90	40.5 [16]	38.61 ± 7.20	39.5 [12.25]	*t* = 0.655 *p* = 0.515	*d* = 0.15
After the program	48.52 ± 5.30	48 [9.25]	33.41 ± 9.83	32.5 [11.75]	*Z* = − 5.823 *p* < 0.001	*d* = 1.91
Test	*t* = − 7.418; *p* < 0.001		*Z* = − 3.577; *p* < 0.001			
Effect size	*d* = 0.97		*d* = 0.72			

^*^“Independent sample *t*-test” (*t*-table value) was used to compare the measurement values of two independent groups for normally distributed data, while the “paired sample *t*-test” (*t*-table value) was applied to compare two dependent groups. For data that did not follow a normal distribution, the “Mann–Whitney *U*” test (*Z*-table value) was used for comparisons between two independent groups, and the “Wilcoxon test” (*Z*-table value) was employed to compare two dependent groups

^**^Cohen *d*

After the program, the mean State Anxiety Inventory score of the adolescents in the intervention group was 32.19 ± 2.75, while in the control group, it was 45.13 ± 4.84. The state anxiety scores of adolescents in the intervention group were found to be statistically significantly lower than those in the control group (*Z* = − 6.669; *p* < 0.001).

Similarly, the mean Psychological Well-Being Scale score of the adolescents in the intervention group after the program was 48.52 ± 5.30, whereas in the control group, it was 33.41 ± 9.83. The difference between the two groups was statistically significant, with higher psychological well-being scores in the intervention group (*Z* = − 5.823; *p* < 0.001).

## Discussion

This study examined the effect of a therapeutic artistic activity program on anxiety and psychological well-being in adolescents with type 1 diabetes mellitus (T1DM). The findings demonstrated that the anxiety scores in the intervention group were significantly lower than those in the control group. Consequently, the H1 hypothesis of this study was accepted. Interestingly, a slight increase in anxiety scores was observed in the control group. This may be attributed to the ongoing challenges of diabetes self-management in the absence of psychosocial support, combined with potential academic or developmental stressors. Moreover, being part of a study without receiving any therapeutic interaction may have created a perception of neglect or increased self-awareness of difficulties, thereby contributing to elevated anxiety. Consistent with these findings, Zamanifard et al. (2022) evaluated the effectiveness of a 6-week painting therapy program delivered via WhatsApp for children with diabetes and reported a significant reduction in anxiety scores within the intervention group [21]. Similarly, studies evaluating the impact of art therapy in adolescents with diabetes have found that anxiety levels significantly decreased, further supporting the present study’s results [13, 16]. The existing literature reinforces that therapeutic artistic activities play a crucial role in alleviating anxiety among individuals with T1DM, highlighting their potential as an effective psychosocial intervention.

The effects of drawing [28], music [29], guided imagery [30], and mindfulness-based programs [31] on anxiety, depression, and metabolic control have been evaluated in adolescents with diabetes. However, these studies have typically examined either artistic activities or cognitive mindfulness-based practices in isolation. In contrast, the integration of artistic activities with cognitive mindfulness-based practices and/or therapeutic approaches (e.g., writing, third-person technique) allows for deeper engagement with the individual’s inner world [32, 33]. This combined approach fosters a holistic framework that emphasizes creativity and self-expression. Particularly during adolescence, the implementation of artistic activities alongside cognitive awareness-based or therapeutic techniques is recommended to enhance mental well-being and improve effective communication [9, 34].

Psychological well-being encompasses key concepts such as self-acceptance, environmental mastery, decision-making, autonomy, and life purpose [35]. Evaluating psychological well-being during adolescence is particularly important, as this developmental stage is marked by identity exploration, autonomy, and the search for meaning. In this study, psychological well-being was assessed in adolescents with type 1 diabetes, and the findings demonstrated a significant improvement in psychological well-being in the intervention group compared to the control group. Consequently, hypothesis H2 of the study was accepted. The existing literature has primarily examined the impact of artistic activities on variables such as anxiety and quality of life [16] as well as self-efficacy [21] in adolescents with diabetes. However, psychological well-being has often been evaluated indirectly in these studies. Research suggests that the positive effects of artistic activities on psychological well-being are linked to factors such as stress reduction, enhanced self-expression, and increased self-confidence [21, 36]. The findings of this study provide valuable insights for the implementation and evaluation of therapeutic programs aimed at improving psychological well-being in adolescents with diabetes.

In conclusion, this study demonstrated that the therapeutic artistic activity program designed for adolescents with type 1 diabetes mellitus (T1DM) effectively reduced anxiety levels and enhanced psychological well-being. The program, developed by considering adolescent developmental characteristics and the psychosocial impact of chronic illnesses such as diabetes, was found to have a high level of effectiveness in both anxiety reduction and well-being improvement.

This study has several strengths. One key strength is its comprehensive evaluation of a program that integrates cognitive, artistic, and therapeutic approaches while addressing both adolescent developmental needs and the specific challenges posed by diabetes. Another strength of this research is that the program content was designed based on the core components of psychological well-being (e.g., autonomy, self-acceptance, meaning, and purpose in life), further reinforcing its theoretical and practical relevance.

However, this study also has some limitations. A primary limitation is that the research was conducted in an outpatient clinic and implemented over a 6-week period, based on the characteristics of adolescent development. Future studies could extend the program duration and assess its impact on metabolic parameters to provide further insights. In addition, the requirement for internet access may limit participation to individuals with adequate digital resources, thereby reducing the generalizability of the findings to lower socioeconomic populations. Another limitation is the potential cultural variability in anxiety perception, which may affect the generalizability of the findings. Another limitation of the study is the lack of long-term follow-up, which restricts conclusions about the sustainability of the observed psychological outcomes. However, it is important to note that the intervention was specifically designed to address state anxiety, focusing on short-term emotional regulation during diabetes management. This focus aligns with prior studies reporting effective implementation of therapeutic art-based programs over 6 to 12 weeks [16, 21]. Future research should explore the long-term effects of such interventions and include trait-level psychological assessments to evaluate more stable emotional outcomes.

## Data Availability

No datasets were generated or analysed during the current study.
